# Assessing Virus Survival in African Swine Fever Virus-Contaminated Materials—Implications for Indirect Virus Transmission

**DOI:** 10.3390/v17010063

**Published:** 2025-01-03

**Authors:** Christina Marie Lazov, Ann Sofie Olesen, Graham J. Belsham, Anette Bøtner

**Affiliations:** 1 Section for Veterinary Clinical Microbiology, Department of Veterinary and Animal Sciences, University of Copenhagen, DK-1870 Frederiksberg, Denmark; asjo@ssi.dk (A.S.O.); anettebotner@outlook.dk (A.B.); 2 Section for Veterinary Virology, Department of Virus & Microbiological Special Diagnostics, Statens Serum Institut, DK-2300 Copenhagen, Denmark

**Keywords:** virus inactivation, ASFV, virus infectivity, qPCR

## Abstract

Introduction of African swine fever virus (ASFV) into pig herds can occur via virus-contaminated feed or other objects. Knowledge about ASFV survival in different matrices and under different conditions is required to understand indirect virus transmission. Maintenance of ASFV infectivity can occur for extended periods outside pigs. Current assays detecting ASFV have inherent disadvantages. Cell culture-based assays are labor-intensive and sensitive to contaminants while methods using qPCR detect ASFV DNA with high sensitivity and specificity, but this may not correspond to infectious virus. Here, we have combined the use of these assays to assess the replication of ASFV within cells and determined the effect of pig feces, straw, wood shavings, and mixed feed on ASFV infectivity. In porcine serum, infectious ASFV survived for at least 60 days at 4 °C, 22 °C, and 37 °C; for two days at 50 °C; one day at 60 °C; and ≤5 min at 70 °C. In the presence of feed, straw, or wood shavings, the survival of the virus wasmarkedly shortened. Samples remained positive in the qPCR assay despite the loss of virus infectivity. Thus, it was possible to distinguish between the presence of ASFV DNA and the survival of the infectious virus.

## 1. Introduction

African swine fever is an important, infectious, hemorrhagic disease of domestic and wild pigs that can have a case fatality rate of up to 100%. It was first described more than 100 years ago [1] and is caused by African swine fever virus (ASFV), the sole member of the *Asfarviridae* [2]. The virus particles are large (ca. 200 nm in diameter) and complex, with a dsDNA genome of 170,000–190,000 bp (depending on the strain), and the multi-layered virions contain over 60 different viral proteins plus about 20 host proteins [3].

Multiple genotypes of the virus are known to exist within Africa [4]. Although initially identified within Africa, there have been several instances of the virus spreading out of Africa. In the 1950s, a genotype-I virus initially caused disease in Spain and Portugal but also spread into Italy (Sardinia) and the Americas (e.g., Cuba and the Dominican Republic) [5]. More recently, a genotype-II virus entered Georgia (in the Caucasus) in 2007 and has spread from there into Asia (including China (the world’s largest pig producer), Vietnam, and South Korea), across Europe, and also into the Americas (Dominican Republic and Haiti), affecting, on a global scale, 62 countries since 1 January 2022 [6]. The disease continues to have a severe economic impact within many of these countries.

Within Europe, the virus has been spreading mainly among wild boar, but spillover into domestic pigs has sometimes also occurred. The virus is readily transmitted via direct contact between infected and uninfected animals [7,8]. However, in addition, transmission via indirect routes in contaminated feed or other objects has occurred (as reviewed in [9]). This is consistent with the “long distance” jumps of the virus, which are believed to have occurred due to human involvement [10,11]. ASFV can be present at high levels within the blood of infected animals [7,8,12], and virus survival seems to be enhanced within blood [13]. Serum has also been reported to have a stabilizing effect on the virus [14,15]. Furthermore, hematophagous insects (e.g., stable flies, *Stomoxys calcitrans*) can take up the virus in blood meals from ASFV-infected pigs [16,17,18] and spread it, via mechanical transmission, potentially into pig farm premises with high biosecurity [19,20]. Note that in eastern and southern Africa, ASFV can be transmitted via a sylvatic cycle involving soft ticks (*Ornithodorous* spp.) and warthogs [21], but the relevant soft tick species are not present within northern Europe; however, even in their absence, extensive spread of the virus, e.g., in the Baltic states, Poland, and Germany, has occurred [11,22].

It is apparent that indirect routes of virus transmission are critically dependent on the stability of the infectious virus in the environment, as well as on the susceptibility of pigs to infection by oral uptake. Typically, the presence of virus within materials, e.g., feed and bedding, is assessed using either infectivity assays in cultured cells, which can be challenging due to the presence of potential contaminants, or by quantitative PCR (qPCR), which does not distinguish between infectious and non-infectious virus.

In previous studies, Dee et al. analyzed the survival of ASFV in feed ingredients and found a half-life of 1.3 to 2.2 days in a variety of different materials. Samples that were still qPCR-positive for ASFV but negative according to virus isolation assays were also tested in a bioassay using intramuscular inoculation of pigs. It was found that this bioassay was able to detect residual virus infectivity in such samples [23]. Analogous results have been observed from the analysis of different dried cured meat products from ASFV-infected pigs. Oral administration of these products to pigs resulted in infection, even when the same products tested negative for infectious virus using cell culture systems [24]. Other studies have identified differences in the minimum infectious dose of the virus when administered to pigs in solid or liquid materials via the oral route [25]. Much lower doses of ASFV were found to be able to initiate infection in pigs when in liquid rather than in solid form.

In this study, assays combining qPCR- and cell culture-based methods to assess the infectivity of samples potentially contaminated with ASFV have been used to assist in determining the influence of common farm materials, which may be contaminated with ASFV, on the survival of the virus under different conditions.

## 2. Materials and Methods

### 2.1. Experimental Design

Closed Eppendorf tubes containing one of four different matrix materials, as well as virus inoculation material, were incubated for pre-determined lengths of time at different temperatures (in the range of 5–70 °C). Two replicate samples (A and B) for each combination of material, time, and temperature were prepared (see Table 1). At the pre-selected times, samples were removed from incubation, quickly put on ice, and stored at −80 °C.

### 2.2. Virus Inoculation Material

A pool of serum from four pigs that had been experimentally infected with ASFV/Podlaskie (as described in [26]) was made and diluted 1:10 with normal (uninfected) pig serum. The undiluted pool had a Ct value of 20.0 in the ASFV DNA qPCR and a virus titer of 6.05 log_10_ TCID_50_/mL, and, after dilution, the Ct value was 23.1. For each sample tube, 500 µL of this diluted serum pool was added to 50 mg straw or 100 mg of the other matrix materials or to an empty tube (positive control).

### 2.3. Incubation Matrix Materials

Four different matrix materials were chosen and collected from a pig farm in Denmark. The materials were barley straw (local production, Denmark), finely chopped to fit into the sample tube; fine wood shavings (Vida strö, Alvesta, Sweden); sow feces; and mixed dry feed (designed for weaning pigs in the age group of 4–6 weeks (optimized by feed consultant)). The mixed and finely ground meal feed consisted of 37.95% wheat (local production, Denmark), 30% barley (local production, Denmark), 17.3% feed mix concentrate (for weaners), 8.85% soya meal (AlphaSoy™ 530, AB Neo, Videbæk, Denmark), 2.9% technical pork fat SF92/15, 2% sugar beet pulp pellets, and 1.5% feed enzyme mix Porzyme^®^ tp 100 (Danisco, Brabrand, Denmark).

### 2.4. Sample Processing

Samples were processed, after incubation, to enable assessment of the presence of infectious virus. To each sample tube, two 3 mm stainless steel balls (Dejay Distribution Ltd., Launceston, UK) plus 500 µL of MEM 10xanti were added (in-house recipe using 500 mL of minimum essential medium with Earle’s salts and L-glutamine (MEM, Gibco, Thermo Fisher Scientific, Waltham, MA, USA) with 1 mL of penicillin, 1.7 mL of amphotericin, and 12.5 mL of streptavidin/neomycin as added antibiotics (Sigma-Aldrich, St. Louis, MO, USA)). The samples were homogenized in a TissueLyser II (QIAGEN, Hilden, Germany) at 25 Hz for 2 min and then centrifuged at 10,000× *g* for 5 min. The supernatants were passed through 0.45 µm sterile filters (Merck Millipore, Burlington, MA, USA) into new Eppendorf tubes.

### 2.5. Virus Nucleic Acid Detection

Aliquots of the filtered supernatants (100 µL) were added to 250 µL MagNA Pure external lysis buffer, and nucleic acids were extracted using the MagNA Pure 96 robot (Roche, Basel, Switzerland) with the DNA and viral NA small-volume kit and an elution volume of 50 µL. The ASFV DNA was detected using qPCR, as described previously [14,27].

### 2.6. Assessment of ASFV Survival

Filtered samples were assayed for the presence of infectious virus via inoculation into primary cells, followed by identification using an immunoperoxidase monolayer assay (IPMA), essentially as described previously [8,28,29]. In brief, the filtered homogenates (100 µL filtrate + 100 µL MEM with 10xanti) were added to the porcine pulmonary alveolar macrophages (PPAM) (1.6 × 10^6^ cells in 1 mL of medium containing 5% fetal bovine serum (FBS)) in NUNC 24-well plates (Thermo Fisher Scientific), incubated for 3 days at 37 °C (with 5% CO_2_), and then frozen (at −80 °C) (first blind passage). Thawed and mixed whole-cell harvests (50 µL) were used to inoculate individual wells in a NUNC 96-well plate of PPAM (with 100 µL of medium with 1.6 × 10^5^ cells per well) for IPMA (as above). After 72 h, cells were fixed and incubated with a polyclonal anti-ASFV antiserum (in-house), followed by a protein A-conjugated horseradish peroxidase (Sigma Aldrich), and then with hydrogen peroxide plus 3-amino-9-ethyl carbazole (Sigma Aldrich) as the chromogenic substrate. Stained cells were identified under the microscope, taking care not to score stained monocytes as positive as these can retain color in the absence of virus (see Supplementary Files S2 and S3). Samples indicating positive results in the IPMA (called “second-passage staining”) were subsequently titrated, when appropriate, using 10-fold dilutions in MEM, starting afresh from the sample filtrates, and stained using first-passage PPAM in the same assay.

### 2.7. qPCR-Based Virus Infectivity Assay

To assess any increase in the level of viral DNA during the blind passaging in PPAM, selected filtered sample homogenates were 12-fold diluted in MEM (100 µL filtrate in a total of 1.2 mL volume as for the passages); the nucleic acids were extracted from 100 µL of this dilution and tested for the presence of ASFV DNA using the qPCR assay (as in Section 2.5). The results were compared with similarly tested extracted nucleic acids present in samples of harvested first-passage material (obtained using 100 µL filtered homogenate with 1.1 mL of MEM with PPAM) after 3 days of incubation at 37 °C. After freezing/thawing, as for the initial homogenate, 100 µL from the cell harvest was used for the nucleic acid extraction. A decrease in Ct value of at least 2 (ca. a 4-fold increase in target DNA) was considered to be positive. Note that for samples scored as negative, an increase in Ct value was often observed in the passaged material, which is indicative of DNA degradation during incubation.

### 2.8. Visualization of Data

Graphs were prepared in GraphPad Prism version 10.2.3 for Windows (GraphPad Software, Boston, MA, USA, https://www.graphpad.com). Final figures containing two or more graphs were subsequently assembled and labeled using BioRender under an academic publication license (https://app.biorender.com/, accessed on 29 December 2024).

## 3. Results

### 3.1. Pilot Studies

**Figure 1:**
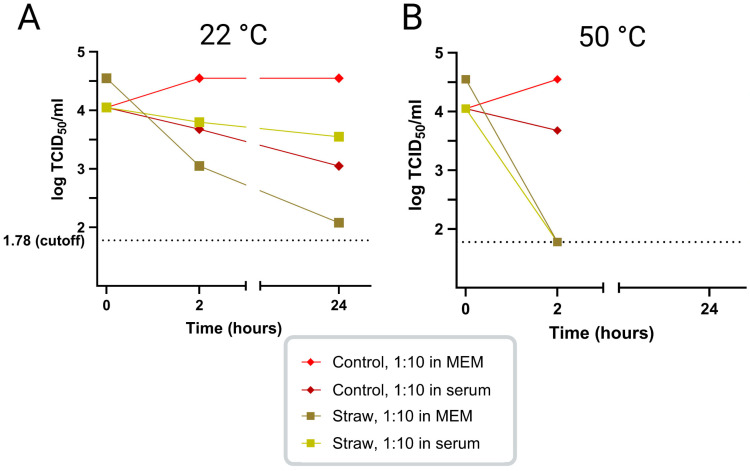
Pilot studies performed with ASFV-containing serum samples with/without straw, diluted 10-fold in either MEM or normal porcine serum, and incubated at 22 °C (panel (**A**)) or 50 °C (panel (**B**)) for the indicated times. The figure was prepared using BioRender [30].

In an initial study, the survival of ASFV infectivity in virus-positive porcine serum, diluted 1:10 in MEM or in ASFV-negative porcine serum, was tested at 22 °C and 50 °C in the presence or absence of straw. At 22 °C, it was found that the virus survived well, for at least 24 h, in MEM in the presence or absence of serum. The addition of straw in the 1:10 dilution of virus-positive serum in MEM seemed to reduce virus survival, but the presence of high levels of serum (100%) seemed to mitigate this effect (Figure 1A). At 50 °C, virus survival was little changed after 2 h but was markedly shortened in the presence of straw; however, no major difference was seen between the virus diluted in MEM vs. serum (Figure 1B). Note that the titer of the control 1:10 in MEM at both 22 °C and 50 °C seemed slightly increased at the 2 h time point, but we consider that this represents normal variation in titration assays. Based on these results, it was decided to perform the subsequent studies using porcine serum from ASFV-infected pigs diluted 1:10 in normal porcine serum instead of MEM.

### 3.2. Analysis of ASFV Survival in Spiked Samples Under Different Conditions

In all the following studies, 10× diluted pooled serum samples from ASFV-infected pigs were added to tubes alone (“positive controls”) or with feed, straw, wood shavings, or pig feces and incubated in closed tubes at different temperatures. Following these incubations, samples were assayed for the presence of infectious virus (in primary cells with detection of ASFV antigen by staining/IPMA) or for the presence of ASFV DNA (by qPCR).

**Figure 2:**
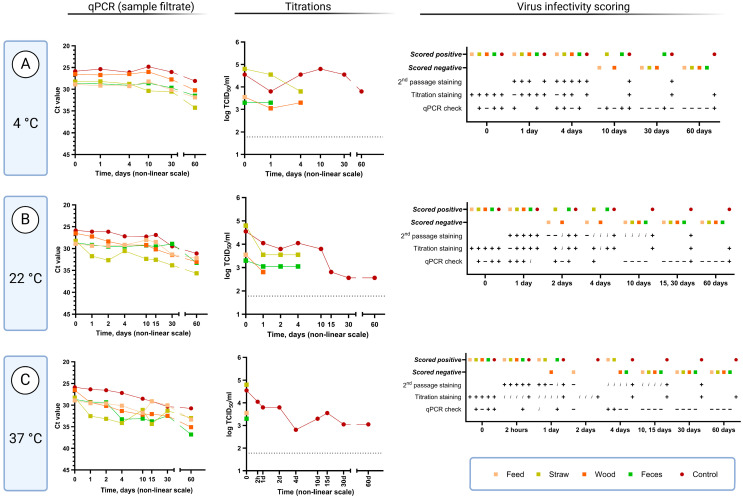
ASFV survival under different conditions. Comparison of sample filtrate Ct values (**first column**) with titers obtained in PPAM-based IPMA (**second column**). Virus infectivity scoring (**third column**) indicates whether virus replication could be clearly defined as positive or negative at the different time points measured. This was achieved by taking into account up to three different assays, as shown in the figure. These were as follows: second-passage staining (fixation and staining of second passages after a blind first passage), titration staining (fixation and staining of dilution series of other first-passage cells for the purpose of titration), and/or qPCR check (by comparing Ct values from qPCR testing of diluted sample filtrate and first (blind) passage material of the same filtrate samples). Samples that were scored as either negative (−), positive (+), or inconclusive (*i*) in these three assays are indicated. Analyses not performed are left blank. Panel (**A**), 4 °C sample incubations: titration of sample filtrates in PPAM was possible up until day 4 after which the samples generated too much background to be read clearly in the assay. The positive control, however, did not have this problem. Panel (**B**), 22 °C sample incubations. Panel (**C**), 37 °C sample incubations: both sample replicates of the positive controls at day 15, 30, and 60 were passaged and assessed, as initial testing of “A replicates” were negative. Following these, tests of B (backup) samples were positive (titration). A few samples at day 2 (straw, wood, and feces) were only tested using passaging and titration and not tested in the qPCR check, and the results were inconclusive. The figure was prepared using BioRender [31].

**Figure 3:**
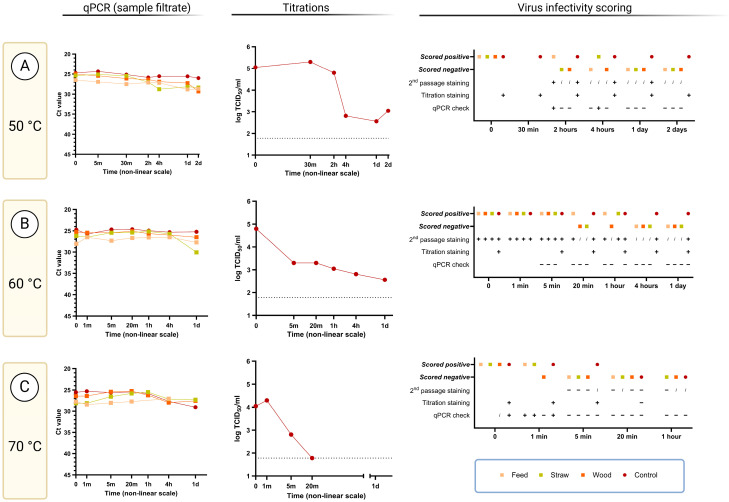
ASFV survival at high temperatures. Comparison of sample filtrate Ct values as determined in the assay for ASFV DNA (**first column**), with titers obtained in the PPAM-based IPMA (**second column**). Virus infectivity scoring (**third column**) indicates whether virus replication could be clearly defined as positive or negative at the different time points measured. This was achieved by taking into account up to three different assays, as listed in the Figure: second-passage staining (fixation and staining of second passages after a blind first passage), titration staining (fixation and staining of dilution series of other first-passage cells for the purpose of titration), and/or qPCR check (by comparing Ct values from qPCR testing of diluted sample filtrate and first (blind) passage material of the same filtrate samples). Samples that were scored as either negative (−), positive (+), or inconclusive (*i*) in these three assays are indicated. Analyses not performed are left blank. Panel (**A**), 50 °C sample incubations: As seen above, no samples at the 5 min timepoint were tested for virus replication. At the 30 min time point, only the positive control was tested (by titration). Panel (**B**), 60 °C sample incubations: All samples shown above were assessed by staining of the second passage (and the controls further assessed by titration of the first passage). Unfortunately, there was complete disagreement between the staining results and the “PCR passage check”. These PCR checks were performed for the feed, straw, and wood samples from 5 min for up to 1 day and for the positive controls at the 1 h and 1 day time points. All PCR checks indicated there was no virus replication in the first passage. Panel (**C**), 70 °C sample incubations: The positive control was found negative in titration at the 20 min time point, meaning that the highest concentration on the plate (filtrate diluted to 10^−1^) was without virus positive cells. This corresponds to a log_10_ (TCID_50_/mL) of 1.78 or below. For virus replication scoring, the samples were all assessed using a “PCR check” of the second passage. The second passages were not stained. Only the positive controls were assessed by titration and staining of the first passage. Note that the positive control at the 5 min timepoint had a low titer, and the “PCR check” also indicated that there was no viral infectivity remaining at this time. The figure was prepared using BioRender [32].

An assay combining cell-based assays with qPCR to assess the presence of infectious ASFV, termed the qPCR infectivity check, was also performed on selected samples to assist with the scoring of samples that were difficult to assess using antigen staining. The assay involved adding the treated samples to PPAM and, following incubation for 72 h, comparing the level of ASFV DNA in the initial filtrate and in the cell harvest. The presence of infectious virus was indicated by increased levels of ASFV DNA (for clarity, a decrease of at least 2 Ct was used to define virus replication; see Supplementary File S1). Using this assay, in combination with staining, the different sample categories could be scored as either negative or positive for the presence of infectious virus (Figure 2 and Figure 3). For all but one of the performed qPCR checks with the samples treated with wood shavings, the infectivity results were negative or inconclusive (decrease in Ct value less than 2). For the other types of samples, qPCR checks were positive at low temperatures and at initial time points but became negative at different points through the course of the experiment, as may be expected.

#### 3.2.1. Virus Survival at 4 °C

At 4 °C, the positive control sample (diluted serum with no added material) was found to contain infectious virus for at least 60 days; this was observed using both the conventional cell staining assays and the qPCR infectivity check assay (Figure 2A, second and third columns). Virus infectivity was also observed and could be titered in the cell-staining assays after incubation for 4 days in the presence of straw and wood shavings but only for 1 day in the presence of feces. Beyond these time points, the samples generated too much background in the cells to be readable. However, using the qPCR infectivity check assay, it was possible to detect virus infectivity in the samples incubated for at least 30 days in the presence of feces, for 10 days in the presence of straw, and for 4 days in the presence of feed. Infectious virus was not detected with the qPCR check at this temperature for any of the tested incubation times in the presence of wood shavings, even though apparent cell staining was observed.

The stability of the ASFV DNA in these samples was also assessed using a qPCR assay on the sample filtrates (Figure 2A, first column). It was found that the level of ASFV DNA detected in the samples was little changed over a period of up to 30 days in the positive control and also in the presence of exogenous materials (feed, straw, wood shavings or feces). Thus, in general, the presence of exogenous materials did not prevent the detection of the ASFV DNA. However, a marked decline in the level of viral DNA detected was apparent in all samples at the next sampling time, which was after 60 days of incubation.

#### 3.2.2. Virus Survival at 22 °C

ASFV, in the positive control, retained infectivity for PPAM, following incubation at 22 °C, for at least 60 days, as judged using the cell staining assay (Figure 2B, second and third columns). In the presence of straw and feces, infectivity could not be detected after more than 4 days of incubation. With feed and wood, infectivity wasonly detected in samples incubated for up to just 1 day. ASFV DNA was readily detected, using qPCR, in all sample filtrates following the 60 days incubation (Figure 2B, first column).

#### 3.2.3. Virus Survival at 37 °C

At 37 °C, the virus infectivity measured in the cell staining assay could be detected in the positive control after 60 days of incubation (Figure 2C, second and third columns). However, even after 30-minutes incubation with any of the exogenous materials tested, it became impossible to read the cell staining titration assay with certainty. In contrast, it was possible to observe infectivity in the qPCR infectivity check assay after incubation of the samples containing feed or straw for 4 days and after 24 h in the presence of feces. The sample containing wood shavings only scored positive up until 2 h of incubation. ASFV DNA could be measured using qPCR in all the samples, even after 60 days incubation (Figure 2C, first column).

#### 3.2.4. Virus Survival at Elevated Temperatures (50–70 °C)

The positive control sample contained infectious ASFV after incubation for 2 days at 50 °C (Figure 3A). However, no clear evidence for the presence of infectious virus couldbe obtained using the cell staining assays in the presence of any of the exogenous materials, except for feed, even after 2 h incubation at this temperature. On the other hand, virus replication could be detected by the qPCR infectivity check assay from the sample containing feed incubated for 2 h and straw for 4 h (Figure 3A, second and third columns). All samples incubated at 50 °C for up to 2 days contained readily detectable ASFV DNA (Figure 3A, first column).

Judging from the cell staining assays, ASFV infectivity was also retained in the positive control sample after incubation at 60 °C for 24 h. Blind passage staining also indicated the presence of infectious virus in the samples with feed and straw for up to 1 h of incubation at this temperature, but with wood, only for 5 min. However, there was no evidence for virus infectivity in any of the selected and tested samples incubated at this temperature, including the control at 24 h in the qPCR infectivity check assay (Figure 3B, second and third column). Note, samples containing fecal material were not incubated at 60 or 70 °C.

Virus infectivity, as judged by antigen staining, was still observed from the positive control after 1 or 5 min incubation at 70 °C but was lost after 20 min incubation (Figure 3C, second and third column). Furthermore, the samples started coagulating after this time point due to the high serum content, which made it impossible to process. In the qPCR infectivity check assay, virus replication could be observed from the positive control, as well as from the samples containing feed or straw after 1 min incubation but not after 5 min. The presence of ASFV DNA was again readily detectable in all samples tested after up to 24 h incubation at 60 or 70 °C (Figure 3B,C, first column).

### 3.3. Results Summary

**Figure 4:**
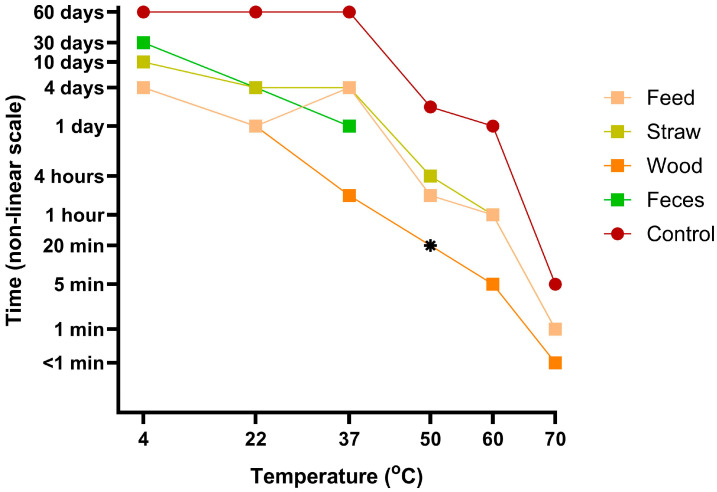
Overview of assessed virus survival. Every point represents the latest time point tested (end point) when infectious virus could be detected (in individual experiments for each temperature). * No data are available from these studies for samples with wood at 50 °C so the end point survival could not be determined.

The latest detected time points for survival of virus infectivity for each sample category, after incubation at different temperatures, are plotted for an overview of the different virus survival experiments (Figure 4).

## 4. Discussion

A novel combination assay has been used here to assess the survival of infectious ASFV following incubations under different conditions. This combination assay includes a so-called “qPCR check” based on measuring the production of ASFV genomic DNA within PPAM (akin to the qPCR check assay described for assessing porcine parvovirus infectivity [33]). Although this system may not appear to be very different from only using staining for ASFV antigens produced within infected PPAM, the qPCR check is less affected by some contaminants/chemicals within the virus samples that cause a high background during the antigen staining procedure. In multiple cases, the qPCR check assay demonstrated its ability to detect infectious ASFV under conditions when it was no longer possible to evaluate infectivity in the antigen staining assay, e.g., for feed and straw samples incubated for 4 days at 37 °C (see Figure 3B, third column). Furthermore, the qPCR check provides a measurable result (Ct difference between qPCR tests of specific nucleic acids in diluted sample filtrate and cell harvest material) in contrast to individual judgement when reading cell staining assays.

The use of these assays in combination also overcomes the major issue with the usual qPCR assays that only detect the presence of the ASFV DNA irrespective of whether this DNA is present within infectious virus or not. It is particularly noteworthy that following high temperature treatment of virus-containing samples, the signal for the presence of ASFV DNA in the sample filtrates was well maintained using the qPCR assay, while the infectivity of the samples was destroyed (see Figure 2 and Figure 3). Thus, the qPCR assays alone are not suitable for assessing the risk posed by materials contaminated by ASFV.

Using these assays, it was possible to assess the duration of ASFV infectivity survival at different temperatures and in the presence of different exogenous materials. Each of the materials tested (feed, straw, wood shavings, and feces) had a negative impact on the survival of ASFV (see Figure 4) throughout the temperature range that was tested. It could be speculated about how these materials may cause these effects. The following considerations may be relevant: (1) the feed contains acidic substances (the medium turned yellow); (2) the straw probably contained molds; (3) the wood produced the most adverse effects perhaps because of tannins or other chemicals reacting with the virus and/or affecting the cells; and (4) the feces were surprisingly benign for the cells and the virus, allowing for the detection of the infectious virus after incubation for up to 30 days at 4 °C in our study. This can be compared to the previously reported survival of up to 8.5 days in feces samples from experimentally infected pigs stored at the same temperature [34].

In stability experiments performed at five different temperatures between −20 °C and 37 °C using plant-based materials, Blome et al. used similar titers and volumes of virus (in EDTA-stabilized porcine blood spiked with virus). However, the matrix materials were used in much larger quantities (e.g., 15 mL of grain and 15 g of straw), and it was difficult to recover infectious virus from the samples, while the presence of ASFV DNA was readily determined [35]. In our studies, on the other hand, we were able to recover infectious virus from our samples for days instead of hours at these temperatures, which could, in part, be due to the lower amounts of matrix materials in each spiked sample. In another stability experiment, in which a larger volume of field crops (20 g wheat, barley, rye, triticale, corn, and peas) were spiked with blood (the ASFV titer of spiking material was 10^6^ HAD_50_/mL), it was also found that it was very difficult to recover infectious virus from dried and heat-treated crops. Hence, even after only 2 h drying at room temperature, infectious virus could not be detected in the spiked feed material using virus isolation in primary porcine cells. In line with our study, ASFV DNA was readily detected in dried and heat-treated virus spiked crops suggesting high stability of ASFV DNA after heat treatment [36]. Interestingly, virus survival at elevated temperatures wasmarkedly increased for control samples (serum) in the current study, compared to the control samples (blood) in the previous study [36]. In blood controls, infectious virus could only be detected for up to 1 h at 50 °C and not after heat treatment at 55 °C [36], while we still detected infectious virus in serum controls after incubation for up to two days at 50 °C, one day at 60 °C, and ≤ 5 min at 70 °C.

The collection of chosen materials and the scale of this study were not meant to constitute an exhaustive investigation into all potential fomites relevant to ASFV transmission. Rather, our study was meant to be the baseline for further studies with an assessment of virus infectivity using sensitive and specific assays. Optimizations could be made in the future to the “qPCR check” assay to enhance accuracy. For example, setting up dilutions of the filtrate for nucleic acid extraction completely in parallel with passaging the filtrate instead of performing it within two separate workflows, as was undertaken here.

It is worth noting that there were several limitations in these studies. Only two replicates were made at each combination of time, material, and temperature, and most often, only one of the replicates was analyzed, while the other was kept as backup. However, it could be argued that since these samples were from time course experiments, the closely connected sample times are semi-replicates. Pilot studies were limited and did not indicate precisely which time points would be optimal to analyze. Therefore, it would be interesting to follow-up with additional samples around the times when changes are seen from positive to negative scoring (end-points, Figure 4), as well as when there was disagreement between the qPCR check and blind passage staining, e.g., for the 60 °C samples (Figure 3B, third column).

All the assays used here for virus infectivity scoring are dependent on replication in primary cells, of which there is a limited supply in our laboratory. Furthermore, the cells could potentially be sensitive to chemical changes that would not necessarily affect the viability of the virus, leading to misinterpretation of virus inactivation when cell viability or health is adversely affected.

The adverse effect of the farm materials on ASFV survival, as measured here, may contribute to the short time window observed for the transmission of the virus from a contaminated environment (e.g., with feces and straw) to newly introduced pigs [29,37]. It has been found that blood can stabilize ASFV [13], and it may be that if blood is shed from ASFV-infected pigs (as sometimes occurs; see [8,38,39], the transmission of the virus will be enhanced, potentially also extending the survival of the virus in the environment. In the current study, the materials and virus were incubated in the presence of porcine serum, which is also known to have a stabilizing and protective effect on the virus [14,15].

It has been shown that infection of pigs with ASFV can occur through ingestion of contaminated materials, such as different feed materials [25]. However, it is also apparent that the inoculation of pigs with virus via the oral route is much less efficient than intranasal inoculation [40]. Furthermore, Niederwerder et al. [25] have reported that the infectious dose of ASFV in liquid form is much lower than the infectious dose within solid feed. These results suggest that the physical nature of the contaminated materials, as well as the level of infectivity within them, will determine whether the infectious virus will be able to cause a new round of infection. The survival of the virus within different materials and under different environmental conditions will clearly contribute to the potential for the indirect transmission of the virus to new host animals.

## Figures and Tables

**Table 1:** Combination of incubation times and temperatures used to assess ASFV survival under different conditions. ASFV was incubated in the presence of serum alone or with one of four different materials, as described in Section 2.3. Samples with feces were not included in the 50, 60, and 70 °C incubations. Light and grey shading alternates between temperatures to ease visual decoding and does not indicate any difference in meaning.

	4°	22°	37°	50°	60°	70°
1 min						
5 min						
20 min						
30 min						
1 h						
2 h						
4 h						
1 day						
2 days						
4 days						
10 days						
15 days						
30 days						
60 days						

## Data Availability

The original contributions presented in this study are included in the article/Supplementary Materials. Further inquiries can be directed to the corresponding authors.

## Supplementary Materials

The following supporting information can be downloaded at https://www.mdpi.com/article/10.3390/v17010063/s1: File S1: Collected data from qPCR checks of passages; File S2: Examples of microscopy images—controls and feed; File S3: Examples of microscopy images—feces, straw and wood.
