# Supplementary figures and images for: Assessing Virus Survival in African Swine Fever Virus-Contaminated Materials—Implications for Indirect Virus Transmission
Source: Viruses. 2025 Jan 3;17(1):63. doi: 10.3390/v17010063 (PMC11769059; doi:10.3390/v17010063)

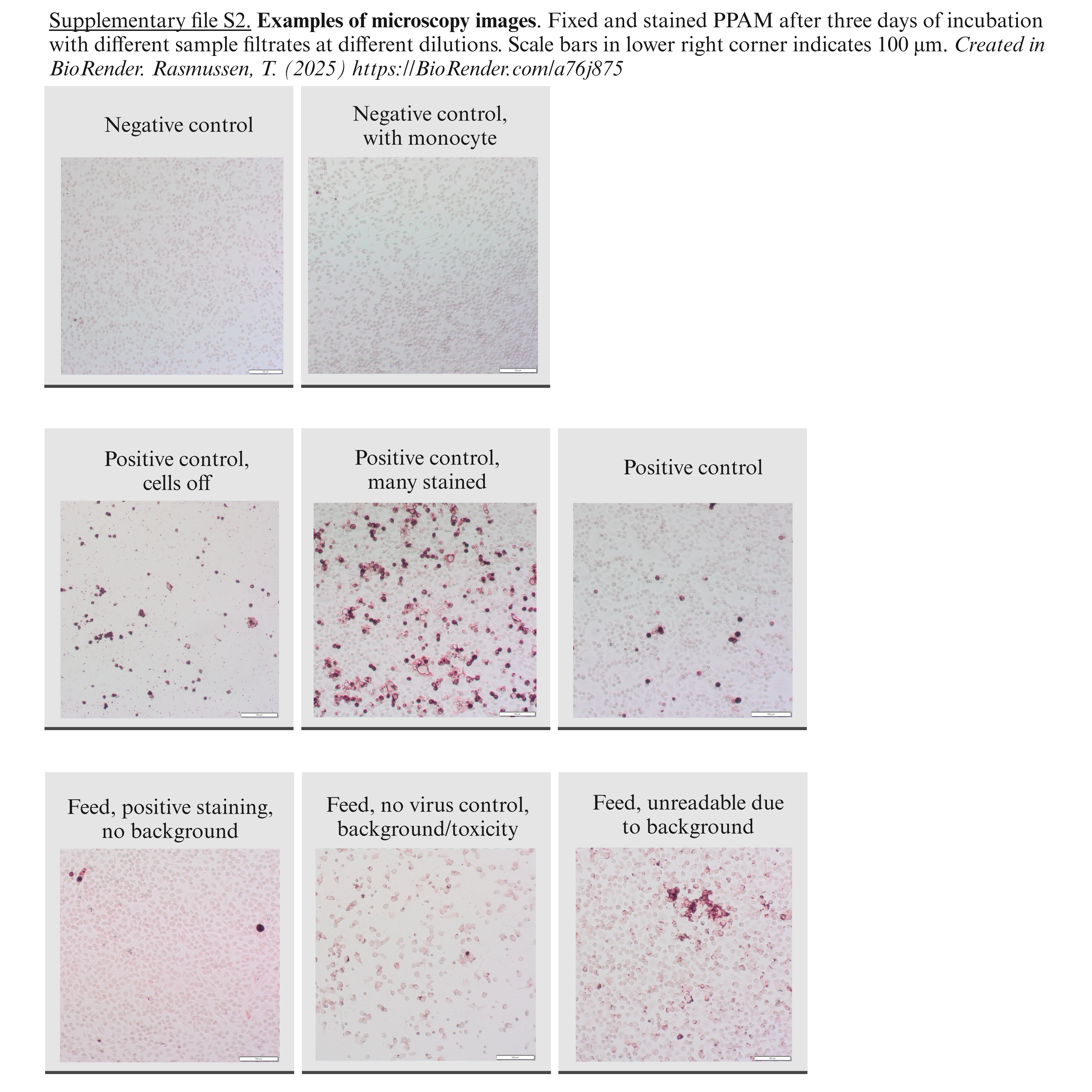

Supplement: Supplementary file 1 [file viruses-17-00063-s001.zip › Supplementary File S2.jpeg]

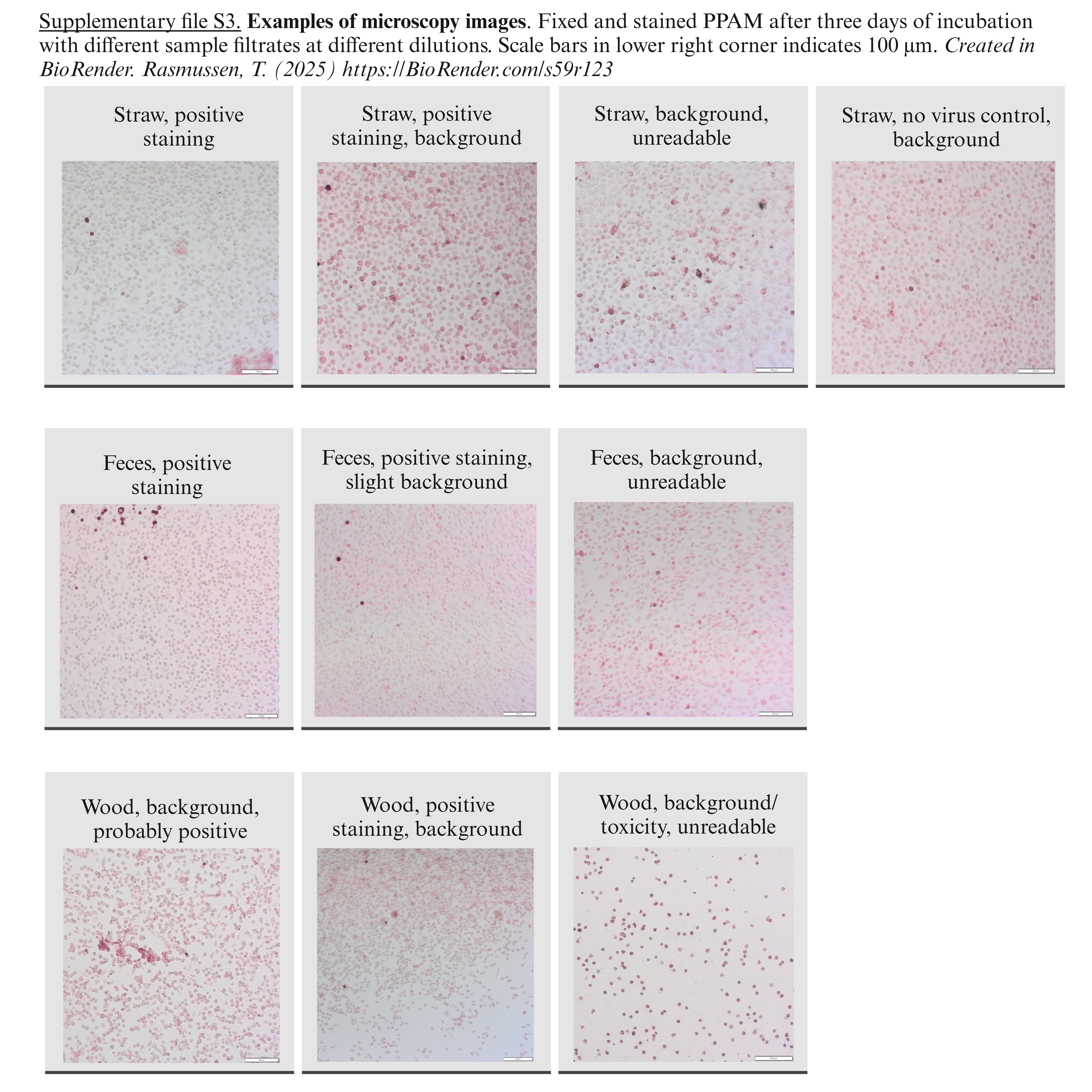

Supplement: Supplementary file 1 [file viruses-17-00063-s001.zip › Supplementary File S3.jpeg]
